# The predictive accuracy of sentinel nodes mapping in the setting of pulmonary metastasectomy

**DOI:** 10.1007/s10585-016-9834-6

**Published:** 2017-01-06

**Authors:** Hyun Koo Kim, Kwanghyoung Lee, Kook Nam Han, Jae Seon Eo, Sungeun Kim, Young Ho Choi

**Affiliations:** 1 Departments of Thoracic and Cardiovascular Surgery, Korea University Guro Hospital, Korea University College of Medicine, 97 Guro-donggil, Guro-gu, 152-703 Seoul, South Korea; 2 Departments of Thoracic and Cardiovascular Surgery, Korea University Anam Hospital, Korea University College of Medicine, Seoul, South Korea; 3 Departments of Nuclear Medicine, Korea University Guro Hospital, Korea University College of Medicine, Seoul, South Korea; 4 Departments of Nuclear Medicine, Korea University Anam Hospital, Korea University College of Medicine, Seoul, South Korea

**Keywords:** Cancers metastatic to the lung, Metastasectomy, Mediastinal lymph node dissection

## Abstract

This is the first study to evaluate the feasibility of mediastinal lymph node dissection (MLND) based on sentinel lymph node (SLN) status during pulmonary metastasectomy. A total of 22 patients (16 men, 6 women; age 63.3 ± 7.01 years) who were candidates for metastasectomy through segmentectomy or lobectomy with MLND owing to cancers metastatic to the lung were enrolled in this study. Radiotracer was administered at the peritumoral region before surgery or soon after initiating surgery. During the operation, the radioactivity of the lymph nodes (ex vivo) was counted with a handheld gamma probe after MLND. Lobectomy was performed in 17 patients, and segmentectomy, in 5 patients. The number of dissected lymph nodes per patient was 14.4 ± 8.69 (range, 5–36). In all patients, the SLN could be detected, and the number of SLNs identified was 2.0 ± 1.15 (range, 1–5) per patient. Lymph node metastasis was identified in 3 of the 22 patients (13.6%), and none of the 3 patients with N1 or N2 disease had false-negative SLNs. SLN identification might be an indicator of whether or not MLND should be performed during pulmonary metastasectomy. However, further large-volume and multi-institutional studies are needed.

## Introduction

The lung is one of the most common sites of distant metastasis of solid tumors because the pulmonary arterial vascular bed is the first filter in the hematogenous route [1]. More than 30% of extra-thoracic malignances give rise to pulmonary metastasis during the course of the disease, and approximately 20% of these cases feature metastases that are confined to the lungs [2].

Pulmonary metastasis represents terminal systemic disease; hence, chemotherapy has been the mainstay of therapeutic intervention in the majority of cases [3]. However, surgical resection of pulmonary metastasis is sometimes necessary for pathological confirmation, and complete surgical excision of all pulmonary deposits is often technically feasible with low morbidity and mortality. Improvement of surgical techniques such as parenchymal-sparing resection, development of more effective chemotherapy regimens, and significantly increased long-term survival as observed in retrospective studies have resulted in pulmonary metastasectomy gradually becoming a standard therapeutic procedure in properly selected patients [1, 4]. The theoretical basis of local treatment, such as surgery with a curative intent, for metastatic tumor is the “cascade-spreading process” of cancer cells to other organs. Even with hematogenous metastasis, there may be a presystemic stage, that is, tumor spread limited to “key-site” organs such as the liver or lung [5, 6].

The incidence of thoracic lymph node involvement in pulmonary metastasis ranges from 5 to 66.3%, and the presence of lymph node metastasis is a significant factor for worse prognosis [7, 8]. The addition of mediastinal lymph node dissection (MLND) to pulmonary metastasectomy remains controversial, because its therapeutic efficacy is insufficient and it is associated with a longer operation time [9]. However, local recurrence seems to decrease when MLND is performed during pulmonary metastasectomy [10, 11]. Additionally, performing lymph node dissection or sampling can reveal unexpected lymph node involvement and provide useful information for postoperative therapeutic decisions [7, 12].

The sentinel lymph node (SLN) is the first pass of lymphatic drainage for cancer cells and is the key lymph node for acquiring information regarding the presence of metastasis from the primary tumor. If metastasis in the SLN is not identified, it is possible to avoid unnecessary lymph node dissection [13]. SLN biopsy has become a standard procedure in the treatment of early stage breast cancer and melanoma as a way to avoid the complications of lymph node dissection. Although the application of SLN mapping to lung cancer surgery has remained controversial, it could be helpful in deciding whether omission of MLND is possible or whether segmentectomy is indicated in patients with clinically node negative disease [14].

The hypothesis of this study was that regional lymph node metastasis in patients with cancers metastatic to the lung occurs in a manner similar to lymph node metastasis in primary lung cancer; hence, SLN identification might be an indicator of whether MLND should be performed or not during pulmonary metastasectomy. However, it is unclear whether SLN identification has the same significance in the setting of hematogenous spread of an extrathoracic primary cancer. This is the first study to evaluate the feasibility of MLND based on SLNs during pulmonary metastasectomy in patients with clinically node negative cancers metastatic to the lung.

## Materials and methods

Candidates for this study included patients who underwent pulmonary metastasectomy with MLND with a curative intent for cancers metastatic to the lung between March 2008 and November 2013 at the Korea University Guro Hospital. After approval by the Ethics Committee of the institution, written informed consent was obtained from all patients in accordance with the Declaration of Helsinki (KUGH14230-001).

For preoperative assessment, each patient underwent thoracic computed tomography (CT) scanning, positron emission tomography (PET/CT), and a pulmonary function test. In principle, the diagnosis and treatment plan for pulmonary metastasis were confirmed by a multidisciplinary tumor board including thoracic surgeons, radiologists, nuclear medicine specialists, and medical oncologists. Pulmonary metastasis was diagnosed on the basis of tissue confirmation through percutaneous needle biopsy under CT-guidance, or on the basis of radiologic confirmation through revaluation after an observation period of at least 2 months after initial diagnosis.

The inclusion criteria in this study followed the basic principles for selecting patients to undergo pulmonary metastasectomy, which were technical resectability, tolerable general and functional surgical risk, control of the primary tumor, and no further extrathoracic metastasis [15]. Patients who had received chemotherapy before pulmonary metastasectomy as neoadjuvant chemotherapy for pulmonary metastasis or repeat pulmonary metastasectomy were not included. In addition, patients with suspicion of metastasis in the hilar or mediastinal lymph nodes as observed on CT or PET/CT were excluded from this study. Mediastinoscopic or endobronchial ultrasonic mediastinal LN biopsy was not performed in any patient. Patients with a single pulmonary metastasis or multiple metastases in the same lobe were included, but patients with multiple metastases in different lobes were excluded.

In our hospital, when a metastasis is located at the peripheral lung and there is no evidence of other pulmonary metastasis, in most cases, we attempt to apply a minimally invasive surgical approach such as video-assisted thoracoscopic (VATS) wedge resection after preoperative localization under CT guidance [16, 17]. MLND is not performed in these patients in order to minimize complications after surgery. However, when the metastasis is located in the central lung, and the resection margin is supposed to be more than 2 cm, segmentectomy is performed. When the resection margin is not sufficient in centrally located metastases, or there is a large tumor or multiple metastases in the same lobe, lobectomy is chosen. VATS major pulmonary resection is performed, regardless of etiology, when the lesion is amenable to anatomic resection and the patient can tolerate single lung ventilation, as determined by preoperative pulmonary function tests in our institution [18, 19]. VATS was not performed in cases of a definite pleural calcification, tight calcification adherent to a pulmonary vessel, thoracic cage deformity, decreased ipsilateral lung volume, a tumor 6 cm or larger, an invasive mediastinal tumor, or the inability to completely resect the lesion. Eventually, patients who underwent segmentectomy or lobectomy as well as complete MLND by VATS or standard thoracotomy were included in this study.

The methods of radioisotope injection and intraoperative SLN mapping were the same as in previous studies [20, 21]. A total dose of 1 mCi of radiotracer (^99m^ Tc-mannosyl human serum albumin; MSA or ^99m^ Tc-phytate) in 0.2 ml was administered at the peritumoral region under chest CT guidance approximately 1 h before surgery in the CT room, or soon after placing ports for video-assisted thoracoscopic surgery or standard thoracotomy during surgery according to the availability of the CT room on the day of surgery. When patients had multiple metastases in the same lobe, radiotracer was injected at the largest lesion. Radioactivity in the lymph nodes was counted after dissection with a handheld gamma probe (Neo2000; Johnson & Johnson, Cincinnati, OH). SLN was defined as any node for which the count was five times the radioactivity of the resected lung tissue with the lowest count. All collected lymph nodes including SLNs were cut into 2 mm slices and diagnosed from formalin-fixed and paraffin-embedded sections stained with hematoxylin and eosin.

### Statistical analysis

The identification rate was defined as the percentage of patients with detected SLNs among the entire analyzed group. The false-negative rate for SLN identification was assessed by the presence of metastatic lymph nodes not identified as SLNs, with the labeled SLNs histologically appearing uninvolved. A *p* value of <0.05 was considered statistically significant. Statistical software (SPSS for Windows, version 22.0; SPSS, Chicago, IL) was used for statistical analysis.

## Results

A total of 22 patients (16 men, 6 women) were enrolled in this study, and their mean age was 63.3 ± 7.01 years (range 52–76). The primary lesions of cancers metastatic to the lung involved the rectum (adenocarcinoma) in 8 patients, the colon (adenocarcinoma) in 8 patients, the kidney (renal cell carcinoma) in 2 patients, the skin (malignant granular cell tumor) in 1 patient, the thigh (sarcoma) in 1 patient, the thyroid (papillary carcinoma) in 1 patient, and the endometrium (adenocarcinoma) in 1 patient (Table 1). The pulmonary metastases were located in the right upper lobe in 4 patients, right middle lobe in 4 patients, right lower lobe in 9 patients, left upper lobe in 3 patients, and left lower lobe in 2 patients. Lobectomy was performed in 17 patients, and segmentectomy, in 5 patients. All patients had a single pulmonary metastasis, except 2 patients who had multiple metastases in the same lobe and underwent lobectomy. The size of the pulmonary metastasis was 2.7 ± 1.90 cm (0.9–7.5).

**Table 1:** Patient characteristics

Characteristic	No.
Sex (Male/Female)	16/6
Age, years	63.3 ± 7.01 (52–76)
Primary lesion (pathology)	
Rectum (adenocarcinoma)	8
Colon (adenocarcinoma)	8
Kidney (renal cell carcinoma)	2
Skin (malignant granular cell tumor)	1
Thigh (sarcoma)	1
Thyroid (papillary carcinoma)	1
Endometrium (adenocarcinoma)	1
Tumor location	
Right upper	4
Right middle	4
Right lower	9
Left upper	3
Left lower	2
Tumor size, cm	2.7 ± 1.90 (0.9–7.5)
Approach	
VATS	17
Thoracotomy	5
Surgery	
Lobectomy	17
Segmentectomy	5

Data are presented as n or mean ± standard deviation (range)

*VATS*  video-assisted thoracoscopic surgery

**Fig. 1:**
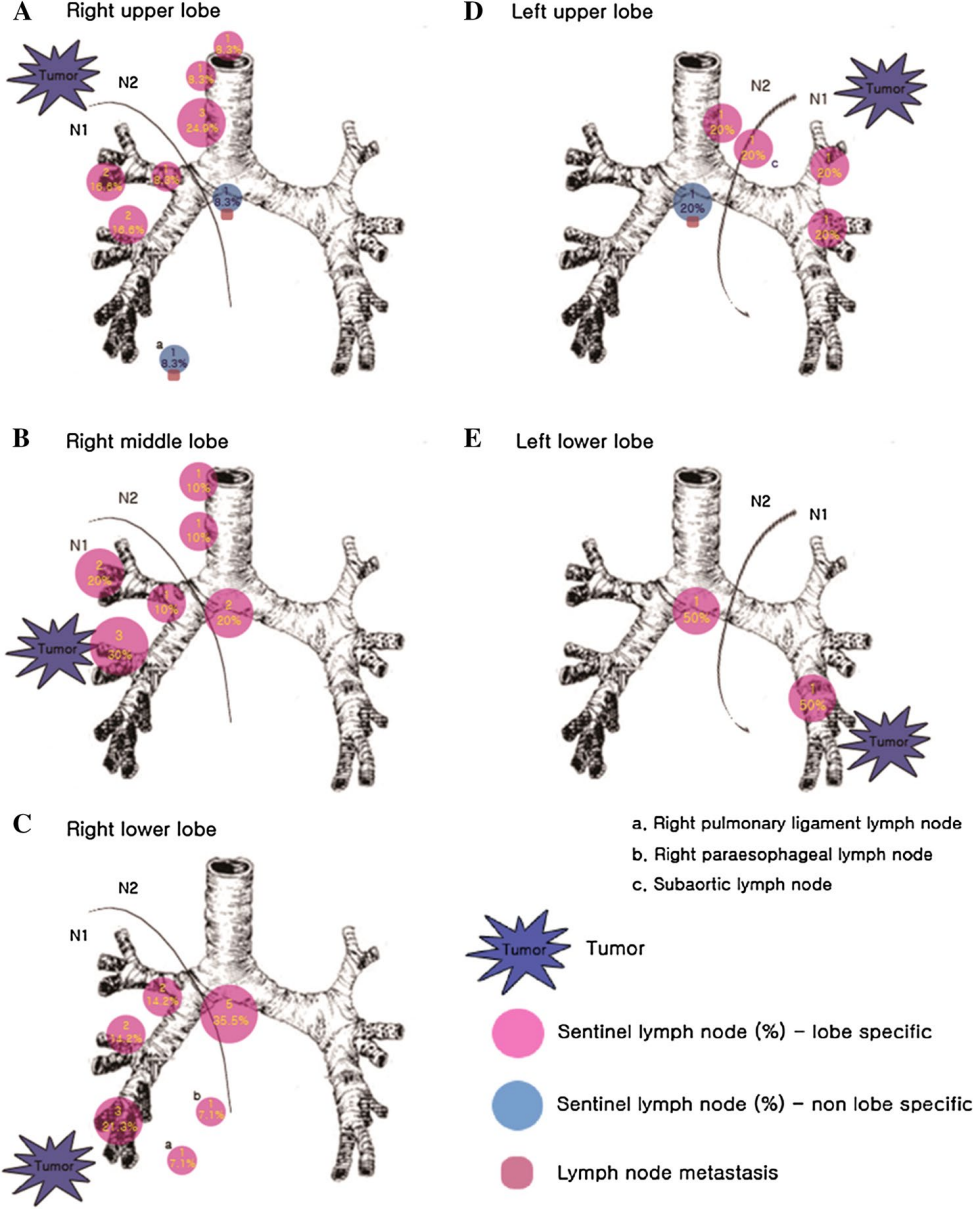
Distribution of sentinel nodes according to tumor location. **a** Right upper lobe, **b** right middle lobe, **c** right lower lobe, **d** left upper lobe, **e** left lower lobe. In patients with a metastatic lung cancer in the right upper lobe, positive sentinel nodes were mostly detected in the paratracheal and interlobar lymph nodes (10/12, 83.3%). Two sentinel nodes (16.7%) were not located in lobe-specific areas such as the subcarinal and inferior pulmonary ligament area. Metastases were found only in non-lobe specific areas

The number of dissected lymph nodes per patient was 14.4 ± 8.69 (range 5–36) (Table 2). In all patients, the SLN could be detected (SLN identification rate, 100%), and the number of SLNs identified was 2.0 ± 1.15 (range 1–5) per patient. SLNs were detected at the mediastinal lymph node station in 17 out of 22 patients (77.3%), and at the hilar lymph node station in 14 out of 22 patients (63.6%). Eight out of 22 patients (36.4%) had SLNs only in the mediastinum, 5 patients (22.7%) had SLNs only in the hilar lymph node station, and 9 patients (40.9%) had SLNs in both the mediastinal and hilar lymph node stations. The distribution of mediastinal SLNs is shown in Fig. 1. In 19 out of 22 patients (86.4%), mediastinal SLNs were located in a lobe specific area. In 3 patients (13.6%), mediastinal SLNs were located in the non-lobe specific area; one patient with cancer metastatic to the lung, involving the right upper lobe, had a SLN at the inferior pulmonary ligament, one patient with metastasis in the right upper lobe had a SLN at the subcarinal area, and one patient with metastasis in the left upper lobe had a SLN in the subcarinal area.

**Table 2:** Results of SLN identification

Characteristic	No.
Dissected LN (No.)	14.4 ± 8.69 (5–36)
SLN detection rate (%)	100 (22/22)
No. of SLNs	2.0 ± 1.15 (1–5)
Metastasis (no. of patients)	3/22 (13.6%)
False-negative SLNs (%)	0

Data are presented as n or mean ± standard deviation (range)

*SLN *sentinel lymph node

Lymph node metastasis was identified in 3 of the 22 patients (13.6%), and skipped metastasis was not noted in any case (Table 3). Among the 3 patients, lymph node metastases were found at lobe-specific areas in 2 patients, but one patient with clinical stage T3N0M0 disease had metastasis in a non-lobe specific mediastinal lymph node as well as in a lobe specific mediastinal lymph node. However, none of the 3 patients with N1 or N2 disease had false-negative SLNs.

**Table 3:** Characteristics of the patients with lymph node metastasis during pulmonary metastasectomy

Sex	Age (years)	Primary cancer	Affected lobe	Tumor size (cm)	SLN station	Metastatic LN station
Female	60	Rectum	Right upper	4	9, 11, 12	11
Male	73	Colon	Right lower	7.5	4	4, 7, 10
Male	56	Colon	Right lower	3.5	7	7, 11

*SLN * sentinel lymph node

No complications were observed during the SLN mapping procedure. There was no mortality or major perioperative morbidity, only minor complications such as prolonged air leak in 2 patients (0.9%) and pneumonia in 1 (0.5%).

**Fig. 2:**
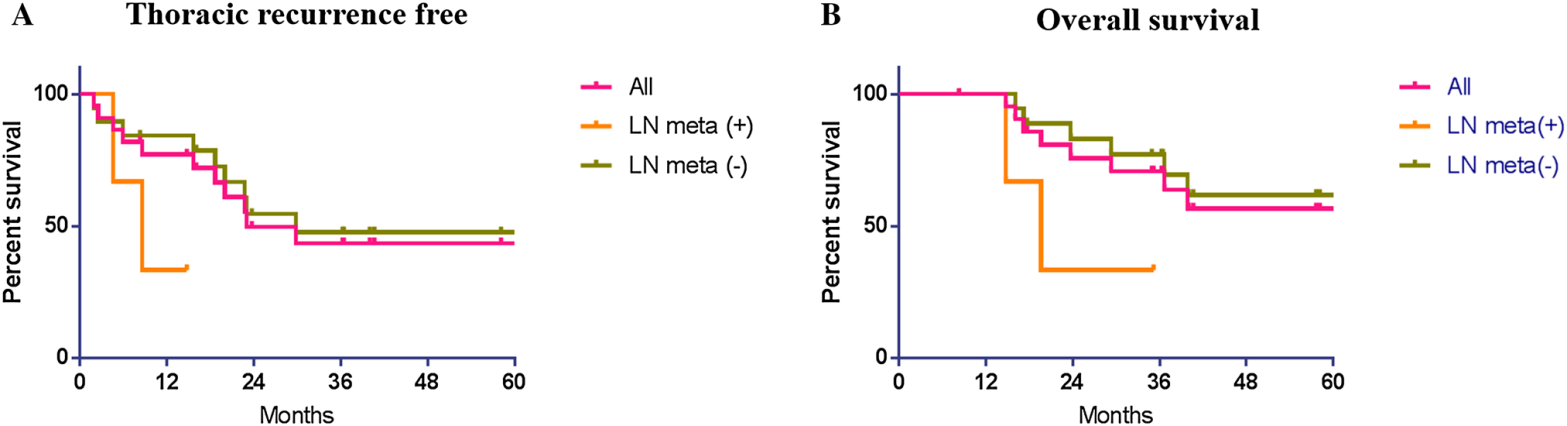
The 3-year thoracic recurrence-free rate and overall survival rate after pulmonary metastasectomy with mediastinal lymph node dissection in clinically node negative patients. The 3 years thoracic recurrence-free rate and overall survival rate was 43.5 and 70.6%, respectively. There were potential differences between pathologically lymph node positive and negative cases

During the follow-up period (41.5 ± 26.1 months), the 3-years thoracic recurrence-free rate was 43.5% in all patients, and there was a potential difference between pathologically lymph node positive and negative cases (54.4% in node negative cases vs. 33.3% in node positive, *p* = 0.07) (Fig. 2). The 3-year overall survival rate was 70.6% in all patients, and there was a potential difference between pathologically lymph node positive and negative cases (77.0% in node negative cases vs. 33.3% in node positive, *p* = 0.05).

## Discussion

MLND may contribute to complete carcinologic control and more accurate patient prognosis, which allows for customized adjuvant treatment even in patients with thoracic lymph node involvement during pulmonary metastasectomy [10]. However, the presence of clinical or pathological lymphadenopathy is still believed to be a relative or absolute contraindication to pulmonary metastasectomy by most thoracic surgeons [22], because the presence of lymph node metastasis is a significant and independent poor prognostic factor in patients with pulmonary metastasis [7, 11, 12]. Moreover, the role of MLND during pulmonary metastasectomy has not been established because its prognostic significance is controversial.

The policy of our hospital is that patients with suspicion of metastasis in the hilar or mediastinal lymph nodes as observed on preoperative CT or PET/CT are not candidates for pulmonary metastasectomy. However, preoperative imaging, such as CT or PET/CT, is limited in determining the presence of metastasis in thoracic lymph nodes, and Seebacher et al., reported that MLND during pulmonary metastasectomy revealed unexpected lymph node involvement in 17% of patients with clinically negative lymph nodes [12]. Kudelin et al. recommend MLND for every patient due to the high prevalence of thoracic lymph node involvement [23]. Given the limitations of these preoperative imaging techniques and the positive impact of subsequent treatment, MLND at the time of metastasectomy appears reasonable in patients suspected to have no thoracic lymph node involvement [12, 13]. However, careful consideration of the need for MLND is required in all patients, because of the increased morbidity and decreased quality of life associated with it. It is thus important for intraoperative decision making regarding MLND to be performed for each patient with pulmonary metastatic disease.

To reduce unnecessary lymph node dissection, many feasibility studies of MLND based on SLN have been conducted for primary lung cancer [24, 25], and we have extensive experience in SLN mapping [14, 20, 26]. We hypothesized that SLN identification might be an indicator of whether or not MLND should be performed during pulmonary metastasectomy if regional lymph node metastasis in cancers metastatic to the lung occurs in a manner similar to lymph node metastasis in primary lung cancer. However, it is unclear whether SLN identification has the same significance in the setting of hematogenous spread of an extrathoracic primary cancer.

In this study, SLN was identified in all cases, and lymph node metastasis was found only in SLNs (no false positives). This result indicates that SLNs identified during pulmonary metastasectomy alone might possibly be metastatic; hence, the decision regarding the performance of MLND could be made during surgery. Furthermore, lymph node sampling or dissection could provide more accurate staging and be used to determine the appropriate adjuvant treatment, but the effects of sampling and dissection on long term survival do not significantly differ [7, 10, 12]. Therefore, obtaining only the SLN during pulmonary metastasectomy might be sufficient to gain accurate information regarding disease status and similar long term survival comparing to MLND. Although lymph node metastasis was identified in 3 patients and skipped metastasis was not noted in any patient (0%) in this study, Pfannschmidt et al. reported that 16.3% (13/45) of patients had mediastinal noncontiguous lymph node spread [27]. However, a large-scale study is needed to address this issue in the future.

This study has many limitations, including the small number of patients, heterogeneity of the primary cancers, the single center experience, and the fact that patients who underwent wedge resection were not included. These limitations should be taken into consideration when interpreting the results of this study.

In conclusion, SLN identification might be an indicator of whether or not MLND should be performed during pulmonary metastasectomy. However, further large-volume and multi-institutional studies are needed to confirm this finding.
